# The Impact of State Preemption of Local Smoking Restrictions on Public Health Protections and Changes in Social Norms

**DOI:** 10.1155/2012/632629

**Published:** 2012-05-10

**Authors:** Paul D. Mowery, Steve Babb, Robin Hobart, Cindy Tworek, Allison MacNeil

**Affiliations:** ^1^Biostatistics Inc., 228 East Wesley Road, NE, Atlanta, GA 30305, USA; ^2^CDC Office on Smoking and Health, 4770 Buford Highway, NE, MS-K50, Atlanta, GA 30341-3717, USA; ^3^ICF International, Denver, CO 80223, USA; ^4^Department of Pharmaceutical Systems and Policy, West Virginia University, Morgantown, WV 26506, USA

## Abstract

*Introduction*. Preemption is a legislative or judicial arrangement in which a higher level of government precludes lower levels of government from exercising authority over a topic. In the area of smoke-free policy, preemption typically takes the form of a state law that prevents communities from adopting local smoking restrictions. *Background*. A broad consensus exists among tobacco control practitioners that preemption adversely impacts tobacco control efforts. This paper examines the effect of state provisions preempting local smoking restrictions in enclosed public places and workplaces. *Methods*. Multiple data sources were used to assess the impact of state preemptive laws on the proportion of indoor workers covered by smoke-free workplace policies and public support for smoke-free policies. We controlled for potential confounding variables. *Results*. State preemptive laws were associated with fewer local ordinances restricting smoking, a reduced level of worker protection from secondhand smoke, and reduced support for smoke-free policies among current smokers. *Discussion*. State preemptive laws have several effects that could impede progress in secondhand smoke protections and broader tobacco control efforts. *Conclusion*. Practitioners and advocates working on other public health issues should familiarize themselves with the benefits of local policy making and the potential impact of preemption.

## 1. Introduction

Preemption is a legislative or judicial arrangement in which a higher level of government strips lower levels of government of their authority over a specific subject matter [1–3]. In the area of smoke-free policy, preemption typically takes the form of a state law or court ruling prohibiting adoption of local smoking restrictions that are more stringent than the state standard. State preemptive laws can also prohibit other local tobacco control measures, such as restrictions on youth access to tobacco products and restrictions on marketing and promotion of these products. Some state preemptive provisions apply to several or all of these domains. In this study, we focus on the impact of state laws that preempt local laws regulating smoking in enclosed public places and workplaces, including restaurants. We set out to document the effect of state provisions preempting local smoking restrictions on three specific outcomes: the number of local smoke-free ordinances in a state, the proportion of indoor employees covered by 100% smoke-free workplace policies, and public support for smoke-free policies in various settings. We selected December 31, 2001 as the time point for our analysis because it provides a relatively large number of data points in the preemption category for analysis. In addition, as of December 31, 2001, no states had enacted comprehensive statewide smoke-free laws.

A broad consensus exists among public health practitioners and tobacco control advocates that preemption has an adverse impact on tobacco control efforts [4, 5]. The *Healthy People 2020* Tobacco-Use Objective TU HP2020-16 seeks the elimination of state laws that preempt stronger local tobacco control laws [6]. Preemptive state laws prevent local governments from taking action to protect residents from the well-documented dangers of tobacco use and secondhand smoke exposure [7]. This is a significant loss, as the strongest and most innovative smoking restrictions—and tobacco control policies in general—have traditionally emerged first at the local level before ultimately being adopted at the state level [8, 9]. The tobacco industry's difficulty in influencing local policy making and the greater influence it is typically able to exercise at the state level have led the industry to lobby forcefully for preemption of smoke-free laws [10–15]. In internal documents, the tobacco industry has expressed concern that strong smoke-free laws will lead to reduced social acceptability of smoking and decreased cigarette sales, while in public the industry has argued, usually indirectly through third-party organizations, that preemption is necessary to ensure a “level playing field” among businesses in different communities, to preserve business proprietors' ability to set their own smoking policies and to prevent local smoke-free laws from adversely impacting restaurant and bar business [8, 13]. In fact, preemptive legislation has often appeared to be a direct response to local smoke-free policy progress. The tobacco industry and its allies have often introduced such legislation shortly after the adoption or consideration of the first local smoke-free ordinances in a state [8, 13].

Successful efforts to impede the adoption of local smoke-free laws have the potential for repercussions beyond reduced protections for nonsmokers. Research, including some conducted by the tobacco industry, has demonstrated that smoke-free policies can also contribute to increased quit attempts and increased success in quitting among adult smokers as well as reduced cigarette consumption among smokers who continue to smoke [13, 16–19]. Tobacco control practitioners believe that preemptive laws have other negative effects, including loss of opportunities for the public debate and education that typically accompany consideration of local smoke-free laws, less vigorous local enforcement efforts, and lower levels of compliance [8, 14, 20–22]. The number of state laws preempting stronger local smoking restrictions increased sharply in the 1990s [14, 15]. In many states, provisions preempting local smoking restrictions were coupled with weak statewide smoking restrictions that contained many exemptions. While some states' preemptive provisions applied only to certain settings (e.g., restaurants in Michigan), allowed local policy making in a limited number of local jurisdictions (e.g., Illinois), or grandfathered local ordinances enacted before a certain date (e.g., Oregon), most states' preemptive provisions were comprehensive in scope, applying to all settings and all local jurisdictions in the state, and effectively blocking any local action in this regard [14].

After peaking in the mid-1990s, the pace of adoption of new state measures preempting local tobacco control policy making leveled off after 1996 [14]. As the pitfalls of preemption became apparent, advocates have pushed for inclusion of explicit non-preemption clauses in state-legislation restricting smoking [1, 20]. In 2002, Delaware became the first state to successfully repeal a provision preempting local smoking restrictions, and eight other states—Illinois, Louisiana, Mississippi, Nevada, New Jersey, Oregon, Iowa, and North Carolina (which rescinded its preemption provisions for some settings, but not for others)—have followed suit, either in conjunction with adoption of a statewide smoke-free law or as a stand-alone action [23]. In other states, such as Kentucky [24] and South Carolina [25] state courts have ruled that state statutes—once widely thought to be preemptive—do not prohibit passage of local smoke-free laws [26]. Although progress is clearly being made toward achieving the *Healthy People 2020* goal of no preemptive state smoke-free laws, as of December 31, 2011, 12 states are still considered to preempt local smoking restrictions in at least one of three major settings (government workplaces, private workplaces, or restaurants) [27].

A comprehensive literature review found just one published study to date that has sought to quantify the impact of state laws preempting local smoking restrictions. Stark and colleagues examined the effect of an Oregon law which preempted local smoking restrictions in conjunction with the establishment of partial statewide smoking restrictions. The law grandfathered in existing local ordinances that were stronger than the state standard. The authors found that nonsmoking restaurant and bar employees working in the preempted communities had elevated levels of a tobacco-specific carcinogen compared to their counterparts working in the grandfathered communities [28].

The current study attempts to quantify the effect of state laws that preempt stronger local smoking restrictions, on a national basis. We examined the number of local ordinances in each state, comparing states with preemption with states that did not have preemption. We also compared the percentage of indoor workers who reported working in a smoke-free worksite, and attitudes about smoke-free laws, between residents of preemption and non-preemption states.

## 2. Methods

### 2.1. Data Sources

We used the 2001-2002 Tobacco Use Supplement to the Current Population Survey (TUS/CPS) to assess the proportion of indoor workers protected by 100% smoke-free workplace policies, public support for smoke-free policies in various settings, and self-reported current smoking status. The TUS/CPS is a nationally representative survey of persons aged 15 years and older conducted by the US Census Bureau and sponsored by the National Cancer Institute and the Centers for Disease Control and Prevention [29]. The national sample is stratified by state, and respondents from all states and the District of Columbia are represented in the sample. The TUS/CPS response rate—which includes response to both the parent CPS survey and the TUS supplement—was 64.0%. Data were weighted to account for probability of selection and nonresponse. Weights were adjusted so that the weighted sample represents the demographic distribution of the US population.

The Centers for Disease Control and Prevention (CDC) State Tobacco Activities Tracking and Evaluation (STATE) System database (http://www.cdc.gov/tobacco/statesystem/), the University of Illinois at Chicago/Robert Wood Johnson Foundation ImpacTeen database (http://www.impacteen.org/), and the American Lung Association's State Legislated Actions on Tobacco Issues (SLATI) database (http://slati.lungusa.org) were used to assess state smoke-free and preemption laws in effect as of the 4th quarter of 2001. The Americans for Nonsmokers' Rights Foundation US Tobacco Control Laws Database (http://www.no-smoke.org/document.php?id=313) of local tobacco control ordinances was used to determine the number of local laws restricting smoking in effect, by state, as of the 4th quarter of 2001. Data on state funding for tobacco control and state cigarette excise taxes were obtained from the CDC STATE System database.

### 2.2. Measures

Self-reported individual-level outcomes examined in this analysis include whether respondents who work indoors are covered by smoke-free workplace policies, and public support for smoke-free policies in various venues, stratified by smoking status. Smoking status was assessed by two questions: “have you smoked at least 100 cigarettes in your entire life?” and “do you now smoke cigarettes every day, some days, or not at all?” Current smokers were defined as respondents who had smoked 100 cigarettes in their lifetimes and who now smoke every day or some days.

The TUS/CPS assessed workplace smoking policy among indoor workers using two questions: “which of these best describes your place of work's smoking policy for indoor public or common areas, such as lobbies, rest rooms, and lunch rooms?” and “which of these best describes your place of work's smoking policy for work areas?” Smoke-free workplaces were defined as workplaces in which smoking is prohibited in both public and work areas. Public support for smoke-free policies in various settings was assessed based on responses to the question: “in 〈venue〉, do you think that smoking should be allowed in all areas, allowed in some areas, or not allowed at all?” The question was asked for six venues—restaurants, hospitals, indoor work areas, bars and cocktail lounges, indoor sporting events, and indoor shopping malls. Support for smoke-free policies in each venue was defined as a response of “smoking should not be allowed at all.” We constructed an index of public support by summing the six responses. Respondents who thought that smoking should not be allowed at all in at least four of the six venues were categorized as supporting smoke-free policies. This index is similar to one used by Gilpin et al. [30].

The presence of a state preemptive provision was measured by creating dichotomous variables for state legislation precluding local smoking restrictions in government worksites, private worksites, and restaurants. Preemption in each of these three locations received one point. Points were summed to create a worksite/restaurant preemption index. We also constructed a preemption score for other public places, including health facilities, recreational facilities, cultural facilities, public transit, malls, public schools, and private schools. Preemption of local smoking restrictions in one or more of these other locations received a score of one point. This score was added to the worksite/restaurant score. Thus, the total preemption index for each state covering worksites, restaurants, and other public places could range from 0 to 4. A preemption score was calculated by multiplying the preemption index by the number of years the preemptive law had been in effect.

A strength of state smoke-free laws index was constructed as the sum of values for state smoking restrictions covering government worksites, private worksites, restaurants, bars, and other public places. For each of these venues, state smoking restrictions were rated as follows: 0: no smoking restrictions, 1: law prohibiting smoking but allowing separately ventilated areas or size exemptions, and 2: 100% smoke-free. Laws that provided for exemptions other than separately ventilated areas or size exemptions were assigned to the “no smoking restrictions” category. Other public places where state smoking restrictions were assessed included hospitals, public transportation, enclosed arenas, grocery stores, shopping malls, prisons, and hotels/motels. For other public places, only the venue with the greatest strength of protection from secondhand smoke was included in the score (values were not summed for each location). The venue-specific scores were summed over the five venues to create the index, yielding a possible range of 0 to 10 for each state's total score.

State funding for tobacco control was calculated as total state funding for tobacco control in fiscal year 2001 divided by the state population. The total includes state cigarette excise tax appropriations for tobacco control, master settlement appropriations for tobacco control, appropriations from other state funding sources, CDC funding, funding for tobacco control activities from the Substance Abuse and Mental Health Services Administration, Robert Wood Johnson Foundation funding, and American Legacy Foundation funding. The excise tax on cigarettes in each state was measured by the inflation-adjusted state cigarette excise tax averaged over the years 1995 to 2001. Taxes were obtained as of the 4th quarter of each year. The state excise tax was averaged over seven years to reflect the cumulative impact that state to state differences in cigarette prices might have on differences in tobacco attitudes and beliefs.

### 2.3. Statistical Analysis

We used a multivariate hierarchical model relating whether the respondent worked in a smoke-free workplace and support for smoke-free policies in various settings to the preemption score for each state, adjusted for other covariates. At the state level, the preemption and smoke-free scores were modeled as continuous variables. US region, state funding for tobacco control, and state cigarette excise taxes were modeled as categorical variables. At the individual level, additional covariates in the model included age, gender, race, and marital status.

## 3. Results

### 3.1. States with Preemption, Venues Affected, and Duration of Preemptive Laws, 2001

As of December 31, 2001, a total of 18 states had provisions preempting local smoking restrictions in at least one of the three major settings considered (Table 1). All but two of these state laws preempted local smoking restrictions in all three major settings as well as in other public places. Only the preemption laws in Michigan and Mississippi applied to fewer than 3 venues. The Michigan law preempted local laws regulating smoking in restaurants; the Mississippi law pertained only to government worksites. These two states did not preempt local smoking restrictions in other public places. Many of the state preemptive laws had been passed during the 1990s. State preemptive laws had been in effect for a median period of 10.5 years. Mississippi's preemptive law had been in effect for the shortest time—only 1 year. Michigan's preemptive law had been in effect for the longest time (18 years), followed by Florida's law (16 years).

### 3.2. Number of Local Ordinances in Preemption And Non-Preemption States

**Figure 1:**
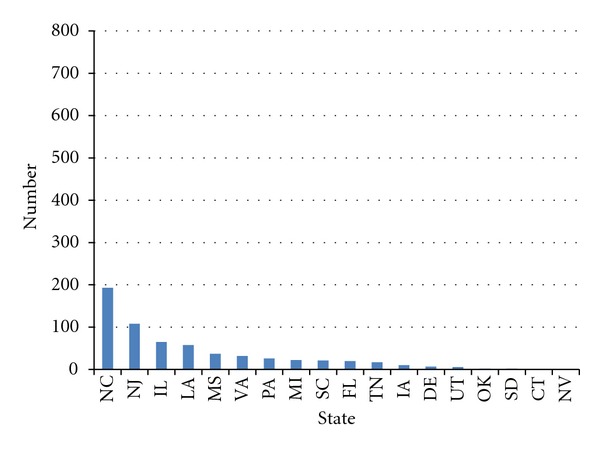
Cumulative number of smoke-free ordinances, preemption states, as of December 31, 2001.

**Figure 2:**
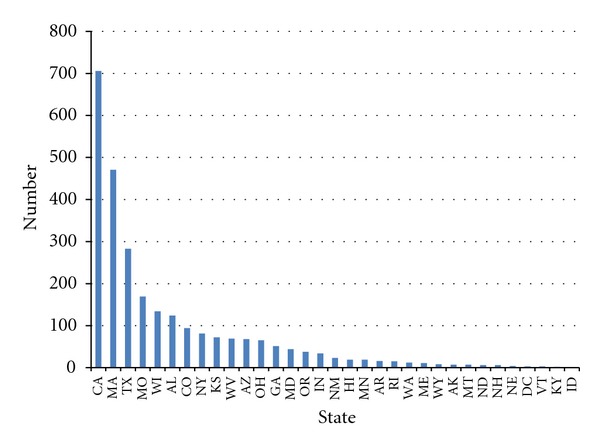
Cumulative number of smoke-free ordinances, non-preemption states, as of December 31, 2001.

By 2001, 3,292 US municipalities had ordinances in effect restricting smoking in one or more public places and workplaces [31]. The mean number of local ordinances of this kind in effect in preemption states was 34.8. The mean number of local ordinances in effect in non-preemption states was 80.8. This difference was not statistically significant (*P* > 0.05) due to the large variability in numbers of local laws within both preemption and non-preemption states (Figures 1 and 2). California had the most local laws in place restricting smoking (706), followed by Massachusetts (471), Texas (283), and North Carolina (193), a preemption state that grandfathered local smoke-free laws adopted before a certain date. All but one state, including preemption states, had at least one local law restricting smoking in effect. Six out of 18 preemption states and ten out of 33 non-preemption states had enacted fewer than 10 local smoke-free ordinances.

### 3.3. State Preemption Score

The state preemption score (Table 1) measures both the number of venues in which local governments are preempted from regulating smoking and the number of years the preemptive law had been in effect as of 2001. The Florida law received the highest (i.e., most restrictive) preemption score of 64, followed by New Jersey (60), Oklahoma (56), and Pennsylvania (52). Mississippi (1) had the lowest score. The mean preemption score for all 18 preemption states was 37.1 (standard error 3.7).

### 3.4. Comparison of Smoke-Free Workplaces and Attitudes about Smoke-Free Policies among Adults Living in Preemption and Non-Preemption States

The percentage of indoor workers who reported working in 100% smoke-free workplaces was higher in non-preemption states than in states with preemption. In a multivariate analysis, the attained significance level for this difference, adjusted for state smoke-free laws, funding for tobacco control, state cigarette excise taxes, US region, and individual covariates, was *P* = 0.06 (Table 2).

Support for smoke-free policies was higher among respondents living in states without preemption than among respondents in preemption states. This difference was observed for current, former, and never smokers. The difference in support for smoke-free policies between non-preemption and preemption state residents was largest among current smokers. In a multivariate analysis adjusted for covariates, this difference was statistically significant among current smokers (*P* < 0.05), but not among former and never smokers (*P* > 0.05).

## 4. Discussion

To our knowledge, this is only the second study to attempt to quantify the effect of state laws that preempt stronger local smoking restrictions and the first study to document the effect of state preemptive laws on a national level and to assess multiple outcomes of these laws.

The most striking finding of the study is that state preemptive laws are associated with reduced support for smoke-free environments in indoor settings among current smokers. (A similar effect was found among former and never smokers, but it was not significant.) In other words, current smokers in states with preemption were less likely to express support for smoke-free environments than their counterparts in states without preemption. State preemption could have this effect by preventing or delaying shifts in social norms that may be generated in part by the discussion, adoption, and implementation of local smoke-free laws.

The discussion and debate that typically occur when communities are considering adopting smoke-free ordinances may raise public awareness regarding the health effects of secondhand smoke and the need for smoke-free policies and contribute to changes in public attitudes regarding the social acceptability of smoking [8, 13]. This discussion also generates news media coverage [32, 33]. Studies have suggested that increased news media coverage of tobacco issues, in turn, is associated with decreases in annual per capita cigarette consumption, decreases in weekly cigarette sales, and increases in adult tobacco use cessation [32, 34–36]. Increased news coverage of secondhand smoke issues may also be associated with increased adoption of local smoke-free laws [37].

In addition to losing opportunities for discussion, residents in preemption states also lose the opportunity to live under smoke-free ordinances. This is a significant loss, as a number of studies have reported that public support for smoke-free environments increases after smoke-free laws go into effect [18, 38, 39]. Studies have found that this effect is especially pronounced among smokers, in part because their baseline levels of support are typically lower than those of nonsmokers [18, 39, 40]. It may be that having the experience of actually living under a smoke-free law dispels concerns about potential adverse effects of such laws and provides firsthand evidence of their benefits. It may also be that people, and smokers in particular, simply adjust to and become accustomed to these laws.

Evidence from a number of states' experiences suggests that the shifts in social norms that occur when smoke-free laws are being considered, adopted, and implemented foster a climate that supports smoking cessation, reduced adult tobacco use, and reduced youth tobacco use initiation [13, 17–19, 41–43]. Evidence also indicates that these shifts lead to increased efforts to reduce secondhand smoke exposure even in settings which are not covered by smoke-free laws, for example, increased adoption of voluntary smoke-free home rules [18, 39]. In fact, the evidence indicates that such changes in public attitudes, which are largely generated by smoke-free laws and other tobacco control policies, are one of the single most important mechanisms through which state and local tobacco control programs reduce tobacco use [44–46]. One of the most significant, although indirect, effects of state preemptive laws may be their denial to state residents of the opportunity to have these experiences during the discussion, adoption, and implementation of smoke-free ordinances, and to undergo the resulting shifts in social norms. This may perpetuate disparities among states in tobacco control policies and tobacco use by freezing local policies and norms in place, thus impeding the efforts of these states to “catch up” with states that have achieved greater progress in reducing tobacco use.

The study also demonstrates that state preemptive provisions are associated with a reduced level of worker protection from secondhand smoke. Indoor workers in states with preemption provisions are less likely to be covered by smoke-free workplace policies than their counterparts in states without preemption.

The implementation of smoke-free laws and smoke-free workplace policies is associated with increased cessation among adult smokers and reduced adult smoking prevalence [13, 17–19]. Several studies have suggested that smoke-free laws and policies are also associated with reduced youth smoking initiation [41, 42]. These effects could operate through several mechanisms, including reduced opportunities to smoke, reduced cues prompting smoking, and shifts in public attitudes regarding the social acceptability of smoking.

This analysis also suggests that state preemptive provisions are associated with fewer local ordinances restricting smoking. This is in keeping with the findings of previous reviews [7, 8, 13].

It should be noted that there are some exceptions to this finding. Some states with preemption have local ordinances in place. This can be due to a number of factors. For example, North Carolina provided a window of opportunity for local jurisdictions to adopt ordinances restricting smoking before the state preemption provision took effect. Other states, such as Illinois, preserved local control in some communities which had already adopted local smoking restrictions before the preemptive state law took effect (i.e., these communities could revisit and strengthen their ordinances). Some states, such as Michigan, preempt local smoking restrictions only in certain venues, while allowing such restrictions in other venues. In other states, such as Florida, local smoking restrictions adopted before the preemptive state law remain in the books, but are not enforced. And in some states local jurisdictions may have passed smoking restrictions, unaware of potential impediments to such action posed by state statutes and legal precedents. However, the data bear out the common sense proposition that the absence of preemption is in most cases a necessary, though not sufficient, condition for the adoption of local smoking restrictions.

This study has some noteworthy strengths, including nationally representative data and control for a variety of state policy and individual-level variables. The study is also subject to several limitations. In particular, because the study is cross-sectional and examines the relationship between state preemption laws and the three outcomes of interest at a single point in time, it cannot establish the causality of the observed associations.

Opponents of smoke-free legislation have not abandoned the use of preemption to impede the adoption of comprehensive local smoking restrictions. In recent years, tobacco control advocates have noted instances in several states of legislation that carves out exemptions for specific venues (e.g., cigar bars and outdoor seating in restaurants) while preempting local governments from restricting smoking in these venues.

In conclusion, this study suggests that state provisions preempting local smoking restrictions affect several outcomes in ways that could impede progress in advancing secondhand smoke protections and broader tobacco control efforts. The issue of the implications of preemption is not unique to tobacco control. Preemption of stronger restrictions at lower jurisdictional levels has surfaced with regard to a number of other public health issues, including alcohol control, and, most recently, menu labeling requirements for restaurants. Because it is somewhat a technical issue and can initially appear to be innocuous, preemption can easily be overlooked, but it can have profound implications. It took tobacco control practitioners and advocates several years to reach consensus on the dangers of preemption—years during which several additional states enacted preemptive laws. It is important that practitioners and advocates working on other public health issues fully understand the benefits of local policymaking and the potential impact of preemption.

There is a need for additional studies replicating the findings of this analysis, especially longitudinal studies. In addition, there is a need for studies exploring the effects of the repeal of state provisions preempting local smoking restrictions on the outcomes we have considered—a type of study that to our knowledge has yet to be attempted.

## 5. Conclusions

This study supports the widely held belief that state provisions preempting local smoking restrictions may impede progress in advancing secondhand smoke protections and broader tobacco control efforts. This underlines the critical importance of preserving local authority in this area.

Preemption of stronger restrictions at lower jurisdictional levels has surfaced with regard to a number of other public health issues, most recently regarding menu labeling requirements for restaurants. It took tobacco control practitioners and advocates several years to reach consensus on the dangers of preemption—years during which several additional states enacted preemptive laws. It is important that practitioners and advocates working on other public health issues fully understand the benefits of local policymaking and the potential impact of preemption in order to avoid repeating this experience.

## Figures and Tables

**Table 1:** States with preemption of local smoke-free laws and preemption score, 2001.

State	Number of major public venues in which local smoke-free laws are preempted (out of 3)^a^	Preemption of local smoke-free laws in at least one other public place?^b^	Number of years preemption in effect	Preemption score^c^
FL	3	Yes	16	64
NJ	3	Yes	15	60
OK	3	Yes	14	56
PA	3	Yes	13	52
IA	3	Yes	11	44
IL	3	Yes	11	44
SC	3	Yes	11	44
VA	3	Yes	11	44
NV	3	Yes	10	40
CT	3	Yes	8	32
LA	3	Yes	8	32
NC	3	Yes	8	32
DE	3	Yes	7	28
TN	3	Yes	7	28
SD	3	Yes	6	24
UT	3	Yes	6	24
MI	1	No	18	18
MS	1	No	1	1

^a^Major venues: government worksites, private worksites, and restaurants.

^b^Preemption of local smoking bans in one or more of these locations: health facilities; recreational facilities; cultural facilities; public transit; malls; public schools; private schools.

^c^Preemption score equals the sum of preemptive restrictions in government worksites, private worksites, restaurants, and other public places (one point for each location) times the number of years the preemptive law had been in effect.

**Table 2:** Percentage of indoor workers with smoke-free workplaces, and percentage of adults who favor bans on indoor smoking, by state preemption status, USA, 2001.

Outcome	State Preemption Status	Percent (95 CI)	(*P* value)^a^
Indoor workers—work in a 100% smoke-free workplace	No Yes	72.4 (72.0, 72.9) 69.1 (68.5, 69.7)	(*P* = 0.06)
Never smokers—favor bans on smoking in indoor places	No Yes	77.8 (77.4, 78.2) 72.6 (72.1, 73.2)	(*P* = 0.12)
Current smokers—favor bans on smoking in indoor places	No Yes	44.1 (43.3, 44.8) 35.6 (34.7, 36.5)	(*P* = 0.02)
Former smokers—favor bans on smoking in indoor places	No Yes	68.7 (68.0, 69.4) 62.8 (62.0, 63.7)	(*P* = 0.06)
Overall—favors bans on smoking in indoor places	No Yes	68.8 (62.8, 74.9) 62.2 (59.8, 64.6)	(*P* = 0.05)

^a^ *F*-test for the hypothesis that average outcomes are the same in preemption and non-preemption states, as estimated from a multivariate hierarchical linear model. In addition to state preemption score, state-level covariates in the multivariate model include smoke-free score, funding for tobacco control programs, state cigarette excise tax, and US region. Individual-level covariates: age, gender, race, and marital status.
